# The evaluation of degeneration of posterior cruciate ligament using CT Hounsfield unit in knee osteoarthritis

**DOI:** 10.1186/s12891-021-04177-w

**Published:** 2021-03-26

**Authors:** Yoshikazu Sumida, Tomoyuki Nakasa, Masakazu Ishikawa, Atsuo Nakamae, Nobuo Adachi

**Affiliations:** Department of Orthopaedic Surgery, Graduate School of Biomedical and Health Sciences, Hiroshima University, 1-2-3 Kasumi, Minami-ku, Hiroshima, 734-8551 Japan

**Keywords:** Degeneration, PCL, Osteoarthritis, CT scan, Hounsfield unit

## Abstract

**Background:**

Posterior cruciate ligament (PCL) degeneration is often seen in knee osteoarthritis (OA); however, there is no established method for its evaluation. The purpose of this study is to investigate whether the Hounsfield unit (HU) using computed tomography (CT) could be a useful scale to evaluate the degeneration of PCL in knee OA.

**Methods:**

Knee OA treated with total knee arthroplasty (21 patients, 21 knees) and non-osteoarthritic knees (21 patients, 21 knees) were retrospectively observed and studied. All PCLs in the knees were analyzed using CT. The PCL in the sagittal section was divided into three regions: proximal, middle, and distal sections. The HU value of the PCL at each area was measured. In osteoarthritic knees, tissues from the PCL were collected and histologically graded. The correlation between the radiological classification by Kellgren and Lawrence and the histological grade was analyzed. The average CT HU values for each degenerative grade were also calculated and compared.

**Results:**

The HU values in OA and non-OA were 70.7 and 88.4 HU (*p* < 0.05) at the proximal region, 75.7 and 85.3 HU (*p* < 0.05) in the central region, and 82.3 and 86.5 HU (*p* > 0.05) in the distal region, respectively. The degeneration of PCL was graded as follows: one, three, and 17 mild, moderate, and severe cases at the proximal portion, and 16, 4, and one mild, moderate, and severe cases at the distal portion, respectively. The radiological classification and the grade of degeneration were not correlated in either the proximal (*r* = 0.047, *p* = 0.84) or the distal (*r* = − 0.21, *p* = 0.35) portions. The HU value was 84.5, 72.1, and 70.6 HU for mild, moderate, and severe grades, respectively (mild versus moderate: *p* < 0.05, mild versus severe: *p* < 0.05, moderate versus severe: *p* > 0.05).

**Conclusions:**

In knee OA, a lower HU value in the PCL indicates the progression of degeneration. The CT HU value could be a useful measurement to predict the grade of PCL degeneration.

## Background

Knee osteoarthritis (OA) is one of the most common diseases encountered in daily practice. Although the pathogenesis of knee osteoarthritis is not yet elucidated, it is known that posterior cruciate ligament (PCL) degeneration is one of the features seen in the process of knee OA [1]. Magnetic resonance imaging (MRI) has been commonly used to evaluate PCL degeneration. However, MRI is only useful for detecting rupture, mucoid degeneration, and ganglion in PCLs; it is not sensitive enough to analyze the quality within the ligaments that have a short echo time [2]. Conversely, computed tomography (CT) is a conventional modality used to assess knee OA. CT is excellent not only for depicting cortical bone and soft tissue calcifications [3], but also for the assessment of soft tissues [4]. It offers better visualization of subchondral bone cysts and osteophytes compared with MRI and radiography, and also helps surgical planning preoperatively [5]. To the best of our knowledge, no studies to date have evaluated PCL using CT. However, it has been reported that CT scans enable quantitative assessment of the soft tissues and materials using the Hounsfield unit (HU) [4]. The HU is also referred to as CT numbers which correspond to the average amount of radiation absorbed by the tissue [6]. The absorption is measured for any given slice relative to water, which is 1 unit. In comparison, the bone is 700 to 3000 units and air 0.001 units. Therefore, we recognized that the HU value could be used to analyze the quality of PCL. We hypothesized that PCL degeneration is relevant to the CT HU value, which provides information about its properties. The purpose of this study was to investigate whether CT HU value could be a useful scale to evaluate PCL degeneration in knee osteoarthritis.

## Methods

### Subjects

This was a retrospective observational study comparing OA and non-OA knees. Twenty-one consecutive OA knees (21 patients) treated with cruciate-retaining total knee arthroplasty (TKA) in our hospital from 2016 to 2018 were retrospectively evaluated. All of the OA knees met the American College of Rheumatology (ACR) Clinical Classification Criteria for Osteoarthritis of the knee [7]. All patients had medial OA knees without any previous history of knee injury, rheumatoid arthritis, or other inflammatory arthritis. For the control group, we included 25 non-OA knees (25 patients) who consulted for the treatment of soft tissue tumors or osteoarthritic ankles in our hospital during the same time period. All of the controls underwent CT scans, which included scans of the knee joints and were required for treatment. The four youngest patients were excluded in order to match based on the sample size and age. The sex distribution was 1 male and 20 females in the OA group and 10 males and 11 females in the control group (*p* < 0.05). The average age was 73.5 years (range, 66–81 years) in the OA group and 73.5 years (range, 58–90 years) in the control group (*p* = 0.94). The average body mass index was 25.8 ± 3.7 kg/m^2^ in the OA group and 22.5 ± 3.4 kg/m^2^ in the control group respectively (*P*<0.05). Based on the radiological classification using Kellgren and Lawrence [8], nine and 12 knees were classified as grade 3 and 4 in the OA group, respectively, and all 21 knees in the control group were classified as grade 0. The anatomical femorotibial angle (lateral angle) was measured on a plain radiograph. The average femorotibial angle was 183.2° ± 8.4° in the OA group and 175.6° ± 1.3° in the control group (*P*<0.05). All patients underwent CT scans preoperatively, and PCLs were evaluated quantitatively using HUs. At the time of TKA, 3 mm × 3 mm × 3 mm large biopsies from the proximal and distal sections of the PCL were performed. All TKA surgeries and biopsies were performed by the same surgeon. Collected tissues were microscopically analyzed and the results were compared with those obtained from the CT scans. The Institutional Review Board (IRB) of Hiroshima University Hospital approved this study, and all procedures were performed with informed consent obtained from all patients.

### Evaluation in CT scans

**Fig. 1:**
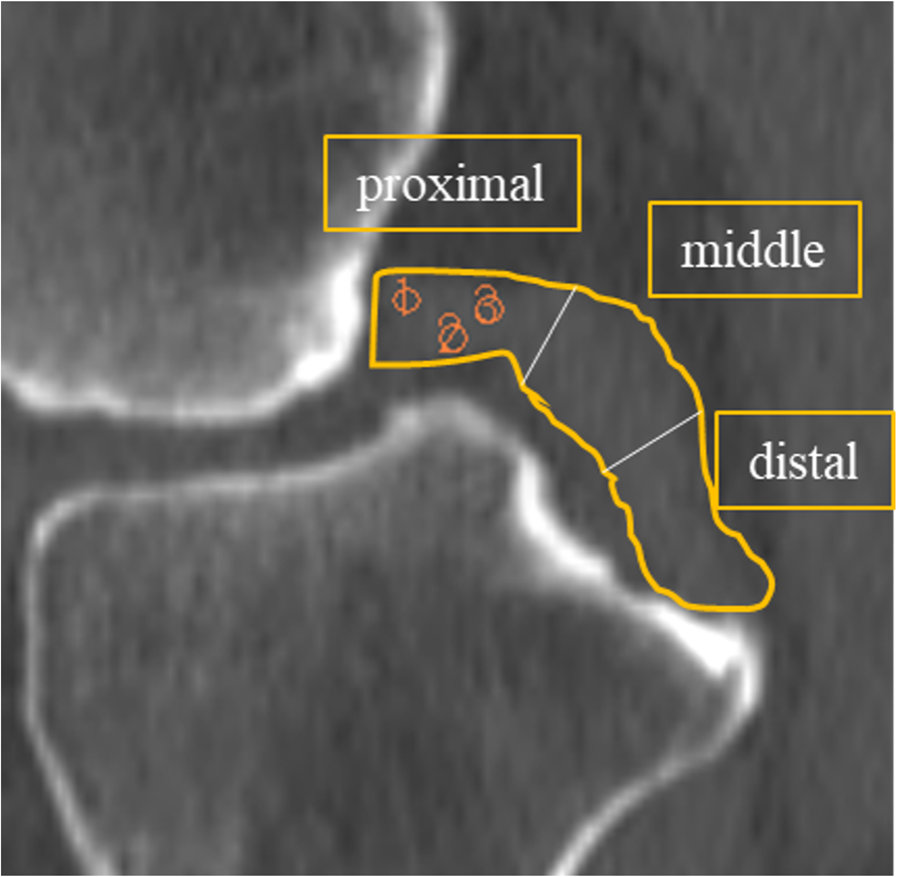
Sagittal image of PCL. PCL is divided into three regions: distal, middle, and proximal. HU in a region of interest (ROI), which was a 3.64 mm^2^ circle, was measured. The average HU from three randomly chosen ROIs was calculated

All patients were scanned with Aquilion One (CANON MEDICAL SYSTEMS CORPORATION, JAPAN). The scanning parameters were as follows: 120 kV, 150mAs, and 1.5 mm slice thickness. Images were analyzed using a workstation, AZE VirtualPlace Ver. 4.7 (AZE, Japan). Patients were asked to fix their knees in extension. All CT scans were taken with the sagittal plane oriented almost parallel to the longitudinal axis of the PCL. An image demonstrating the center of the PCL was selected, and the PCL was divided into three parts: proximal, middle, and distal sections (Fig. 1). We measured the HU value in a region of interest (ROI), which was a 3.64 mm^2^ circle, in PCLs. The measurement was performed in three random plots in each section, and the average HU value from these three plots was calculated. The obtained HU values were compared between the two groups.

### Microscopic analysis of PCLs

**Fig. 2:**
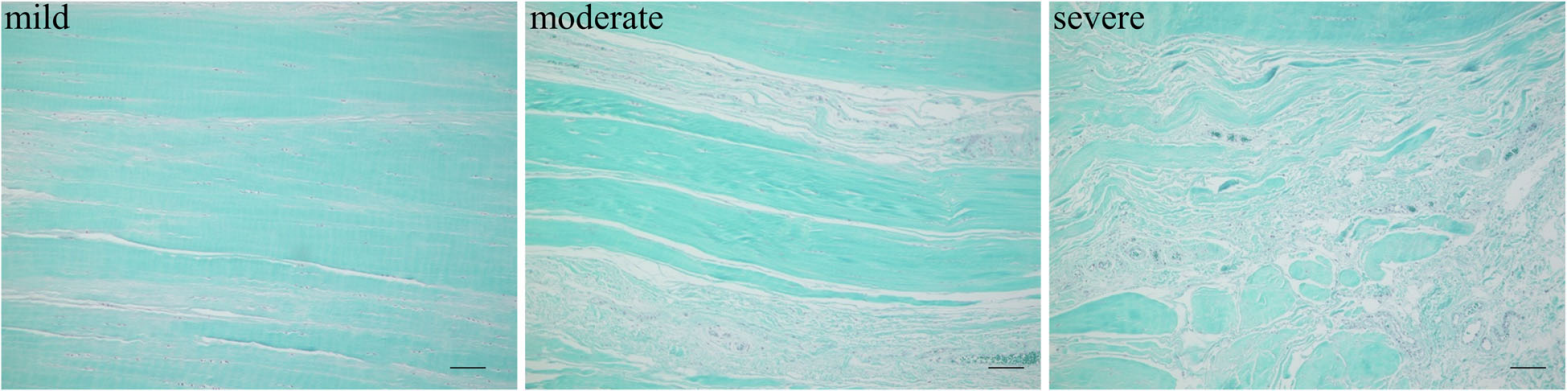
Collagen fibers in PCL stained with Safranin-O fast-green. The grade of collagen fiber impairment was classified as follows: mild, impairment of less than 20% of the collagen fibers; moderate, impairment of at least 20% and less than 50% of the collagen fibers; and severe impairment of more than 50% of the collagen fibers. The scale bar indicates 100 μm

At the time of TKA, samples were carefully resected from the proximal and distal portions of the PCLs. The samples were fixed in 4% paraformaldehyde in phosphate-buffered saline (Wako Pure Chemical Industries Ltd., Osaka, Japan) immediately after collection. Twenty four hours later, they were embedded in paraffin and cut into 5 μm-thick sections. The sections were stained with Safranin-O fast-green and evaluated microscopically. We evaluated the specimens at 100× in the examination of ten fields according to the method described in previous studies [9]. The grade of collagen fiber impairment was classified as follows: mild, impairment of less than 20% of the collagen fibers; moderate, impairment of at least 20% and less than 50% of the collagen fibers; and severe impairment of more than 50% of the collagen fibers (Fig. 2). For statistical analyses, scores ranging from 1 to 3 were assigned as follows: 1 = mild, 2 = moderate, and 3 = severe. In the OA group, the distribution of the degeneration was analyzed both in the proximal and distal portions. The correlation between the radiological classification by Kellgren and Lawrence and the grade of collagen fiber impairment was analyzed in both portions. The average CT HU values for each degenerative grade were also calculated and compared.

### Statistical analysis

Data were analyzed using the Statistical Package for Statcel 4 (OMS, Saitama, Japan). All measured values were expressed as means ± standard deviation (SD). The Mann-Whitney and Kruskal-Wallis tests were used for comparisons between two groups and three groups, respectively. Spearman’s nonparametric correlation coefficients were used to assess the strengths of the bivariate associations. Fisher’s exact test was used to analyze differences in the sex distribution between the two groups. *P*-values < 0.05 was considered statistically significant.

## Results

**Fig. 3:**
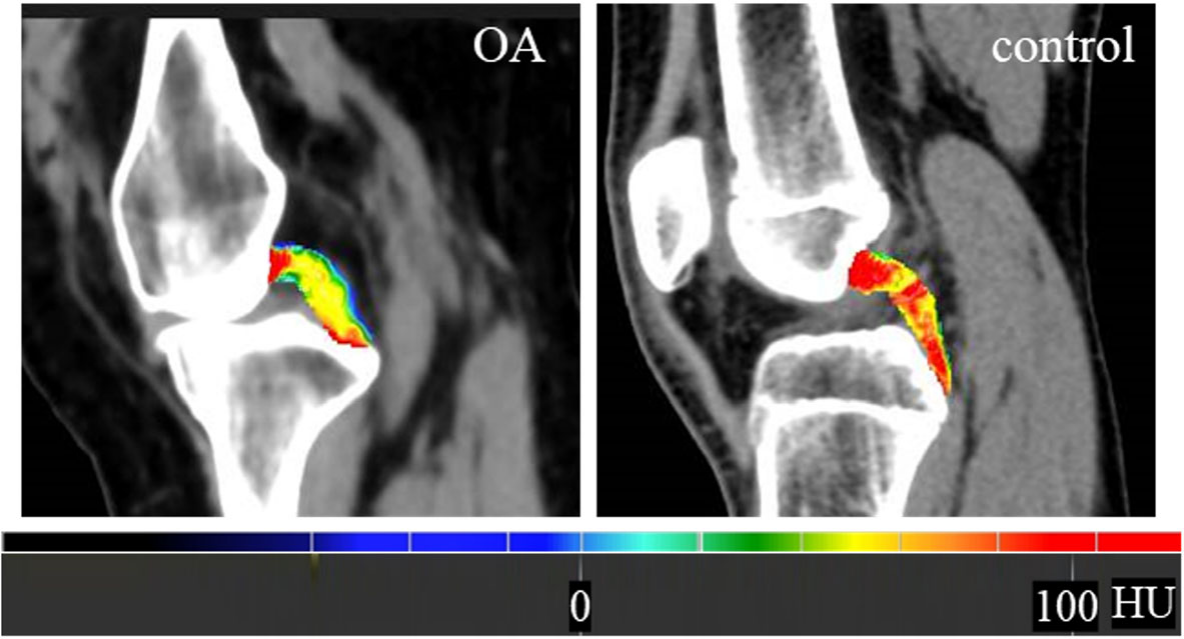
Quantitative comparison of CT HU value maps between the OA and control groups. The image of OA shows a lower CT HU value in the PCL

Quantitative comparison of CT HU value maps demonstrated that the OA group had a lower HU value (Fig. 3). At the proximal portion, the CT HU value was 70.7 ± 9.8 HU and 88.4 ± 4.7 HU in the OA and control groups, respectively; this difference was significant (*P*<0.05). At the middle portion, it was 75.7 ± 11.7 HU and 85.3 ± 4.5 HU in the OA and control groups, respectively; again, this difference was significant (*P*<0.05). At the distal portion, it was 82.3 ± 14.1 HU and 86.5 ± 5.1 HU in the OA and control groups, respectively; however, this difference was not significant (*P* > 0.05) (Table. 1).

**Table. 1:** The average CT HU value ± standard deviation of PCL from each region

	Control	OA
**Proximal**	88.4 ± 4.7	70.7 ± 9.8**
**Middle**	85.3 ± 4.5	75.7 ± 11.7**
**Distal**	86.5 ± 5.1	82.3 ± 14.1

The value was significantly lower in the proximal and middle portions of the PCL. (**p < 0.01, Mann–Whitney test)

**Fig. 4:**
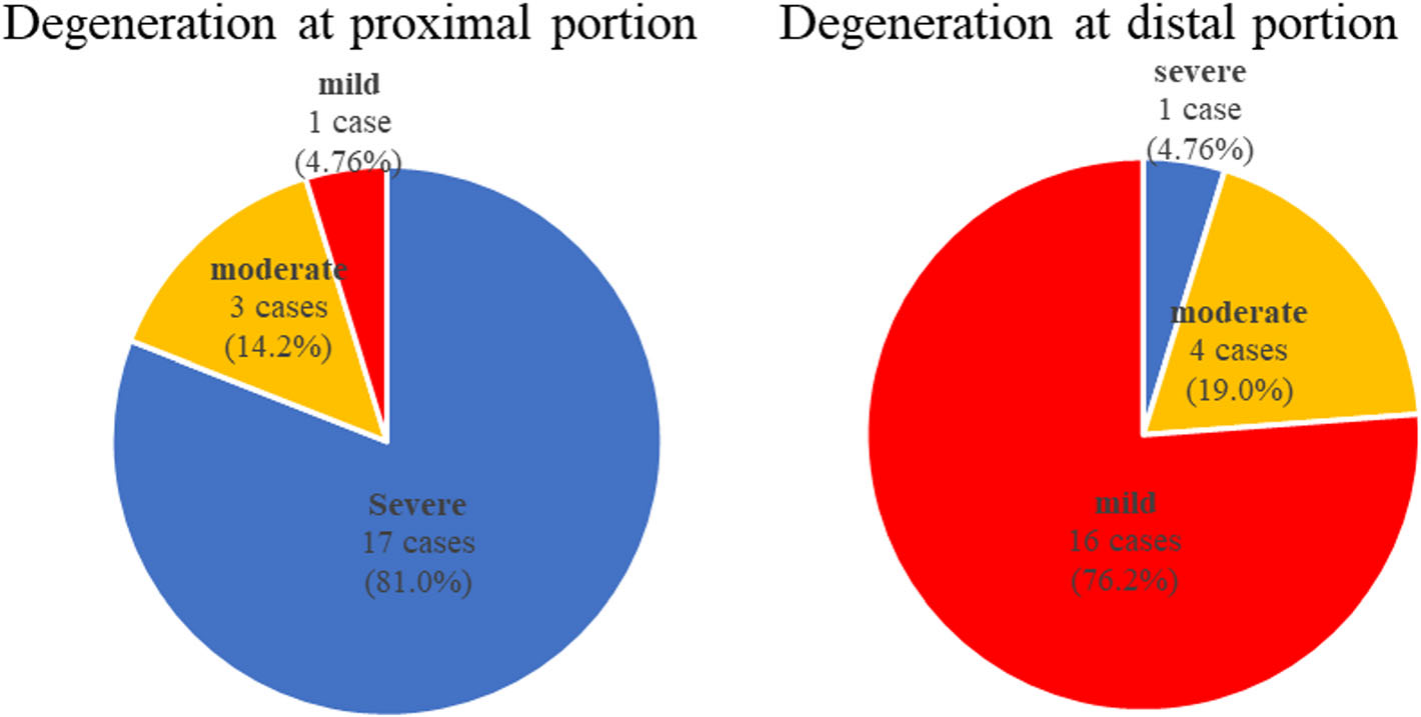
Cases (number and proportion) with different grades of degeneration at the proximal and distal portions. More severe cases were observed at the proximal portion compared with the distal portion

In the OA group, microscopic analysis of the PCL in OA knees revealed severe degeneration in 17 (81.0%) cases, moderate degeneration in three (14.2%) cases, and mild degeneration in one (4.76%) case in the proximal portion, and severe degeneration in one (4.76%) case, moderate degeneration in four (19.0%) cases, and mild degeneration in 16 (76.2%) cases in the distal portion (Fig. 4). None of the samples demonstrated mucoid degeneration presenting the distinct deposition of the mucoid matrix.

The radiological classification by Kellgren and Lawrence and the grade of collagen fiber impairment were not correlated in both the proximal (*r* = 0.047, *p* = 0.84) and the distal (*r* = − 0.21, *p* = 0.35) portions.

**Fig. 5:**
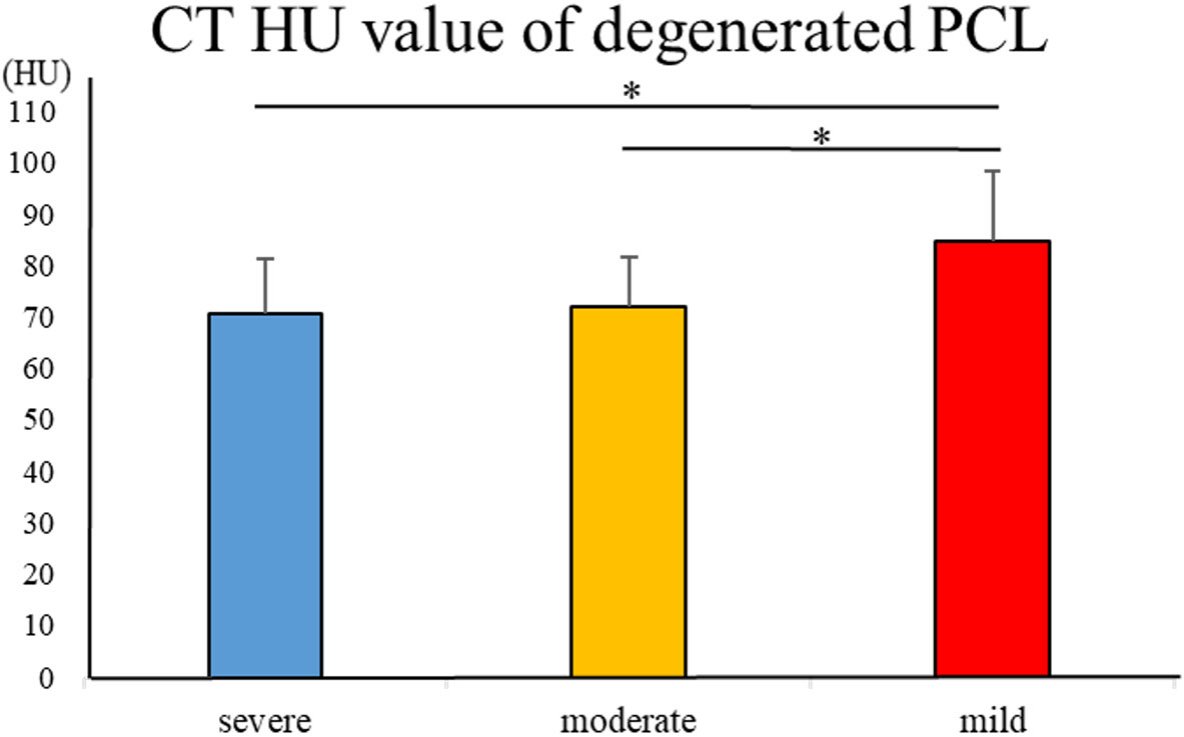
The CT HU values for PCL with different degenerative grades. The CT HU value of mild degeneration was significantly higher than that of the others (mild versus moderate: *p < 0.05, mild versus severe: *p < 0.05, moderate versus severe: *p* > 0.05, Kruskal-Wallis test). The error bar shows the standard deviation

The CT HU values of PCL with severe, moderate, and mild degeneration were 70.6 ± 10.7 HU, 72.1 ± 9.50 HU, and 84.5 ± 13.7 HU, respectively. The CT HU value for mild degeneration was significantly smaller than that for severe and moderate degeneration (*P* < 0.05) (Fig. 5).

## Discussion

The most important finding in this study is that we demonstrated that the HU value of the PCL could reflect the grade of degeneration. Additionally, we revealed that PCL degeneration in knee OA was more severe at the proximal portion.

In our study, the HU value in the PCL with mild degeneration was 84.5 HU; this was significantly higher than that for severe and moderate degeneration (70.6 HU and 72.1 HU, respectively). To our best knowledge, this is the first study to demonstrate the HU value of the PCL in knee OA. No previous studies have reported on the utility of the HU value for the evaluation of PCL or the anterior cruciate ligament (ACL). Willner et al. revealed the typical HU values for various tissues in the literature: tendon; 101.6 HU, muscle; 39.4/40.4 HU, adipose; − 30.6/− 66.6 HU [10]. However, they did not mention the HU of ligamentous tissue. Chikui et al. suggested that myxoid and pleomorphic sarcomas could be easily differentiated from lipomas because the CT values would be higher than those of adipose tissue [11]. In another study, Spruit et al. showed that the HU value of bone grafts in intervertebral metal-cage contents increased in the postoperative period, which demonstrated biological activity and a change in the bone mineral content of the tissue [12]. Barber et al. evaluated the long-term in vivo degradation of a biocomposite interference screw with the HU value and confirmed its osteoconductivity [13]. They also analyzed the density of the synthetic multiphase implant for donor site autologous osteochondral transplantation in another study [4]. The density declined over time to that of the fibrous scar, which revealed no evidence of bone ingrowth, osteoconductivity, and ossification of the implant. According to these previous reports, the HU value using CT scans could provide objective and quantitative evaluation of soft tissues and help in the prediction of the tissue properties.

Concerning PCL degeneration, Kleinbart et al. investigated the histological changes in PCLs from osteoarthritic knees and age-matched normal knees. They described that PCLs in osteoarthritic knees showed distinct histologic degenerative changes compared with age-matched normal knees [14]. In another report, Levy et al. revealed a lack of correlation between PCL degeneration and age [15]. They also described that PCL degeneration was detected even in early stage OA knees and was associated with ACL and cartilage damage. Similarly, Aggarwal et al. concluded that ACL appearance and insufficiency, and erosion in the lateral tibiofemoral compartment were predictors of PCL generation [1]. They considered that PCL generation could be caused by instability due to ACL rupture and subsequent wear of the lateral tibiofemoral compartment in the arthritic process. These previous studies might provide an explanation as to why PCL in OA knees had more severe degeneration compared with non-OA knees in our study. We could not identify a correlation between the radiological classification by Kellgren and Lawrence, or the grade of PCL degeneration. A possible reason for this is that the radiological classification is only evaluated in the coronal plane and it does not sufficiently reflect the instability, which potentially affects PCL degeneration.

Another finding in this study was that PCL in knee OA showed more severe degenerative changes in the proximal portion with a lower HU value compared with that in the distal portion. Levy et al. demonstrated that the first changes observed in the PCL were fiber disorganization, mucoid, and chondroid metaplasia [15]. The study by Kumagai et al. indicated that chondroid metaplasia is associated with the progression of degeneration in human ligaments [16]. The lower HU value in the proximal portion of the PCL could reflect those histological changes. They evaluated PCL by scoring inflammation, mucoid degeneration, chondroid metaplasia, and cystic changes other than orientation of collagen fiber. Conversely, Viidik et al. demonstrated that changes in collagen fibrils affect the biomechanical properties of the ligament [17]. Thus, we evaluated the degeneration only in terms of collagen fiber impairment, because we believe that the impairment of collagen fibers is the most effective factor in terms of ligamentous function, and that it should be evaluated microscopically. More severe degenerative changes were detected in the proximal portion in this study; however the reason for this is unclear. Although Levy et al. and Kumagai et al. described PCL degeneration with histological evaluation, they analyzed the tissue resected only from the middle portion and proximal one-third of the PCL, respectively [15]. To our knowledge, no studies have demonstrated the distribution of the emergence of degenerative changes in PCL. It has been reported that ACL degeneration occurs in the proximal portion [18]; however, the mechanism and pathogenesis of PCL degeneration is still unknown and should be elucidated in the future.

Prediction of the quality of PCL using the CT HU value could be more beneficial for both surgeons and patients undergoing TKA. There is controversy regarding whether PCL should be retained or sacrificed at the time of TKA for knee OA [19]. Even if the macroscopic appearance of PCL is normal, degenerative changes are observed microscopically [20]. It is ideal to know the grade of degeneration in PCL preoperatively and choose an adequate device for each patient, which would bring greater benefits to both patients and surgeons. Considering the fact that MRI is not useful enough to analyze the quality within the ligaments that have a short echo time [2], the measurement of the CT HU value could be a valid method to provide us with more details about the ligament property. Although further studies are required, evaluation using the CT HU value could be applied not only for PCL but also for other soft tissues such as tendons and cartilage. For instance, in case of cruciate ligament reconstruction with the hamstring tendon, surgeons could evaluate the property of the reconstructed ligament with the CT HU value postoperatively, which could help them to make decisions about when to allow patients to return to play sports. Evaluation using the CT HU value could provide us with more information about the status of soft tissue.

There are some limitations to this study. First, the sample size was small; a larger population should be evaluated in both groups in future studies. Second, the difference in the number of males and females included in the OA and control groups was significant. More women were included in the OA group and more men in the control group. The influence of sex was thus not considered in this study. Third, one sagittal image from CT scans might not be enough to correspond to the histological result of the obtained sample. Lastly, bone or soft tissue tumors might have affected the properties of PCL. However, they were the best control group that could be obtained because it is ethically challenging to require healthy people to undergo CT scans considering the exposure to radiation.

## Conclusions

In this study, we revealed the utility of the HU value using CT scans for the evaluation of degenerated PCL in knee OA. The value decreased as degenerative changes proceeded. The CT HU value could be a useful measurement to predict the grade of PCL degeneration.

## Data Availability

The datasets used and analyzed during the current study are available from the corresponding author upon reasonable request.

## Abbreviations

OA: Osteoarthritis
PCL: Posterior cruciate ligament
MRI: Magnetic resonance imaging
CT: Computed tomography
HU: Hounsfield unit
ACL: Anterior cruciate ligament
